# Estimating the impact of pneumococcal conjugate vaccines on childhood pneumonia in sub-Saharan Africa: A systematic review

**DOI:** 10.12688/f1000research.25227.2

**Published:** 2020-11-09

**Authors:** Chukwuemeka Onwuchekwa, Bassey Edem, Victor Williams, Emmanuel Oga

**Affiliations:** 1 Institute of Tropical Medicine, Antwerp, Antwerp, 2000, Belgium; 2 Medical Research Council Unit The Gambia at London School of Hygiene and Tropical Medicine, Serekunda, The Gambia; 3 School of Public Health, Faculty of Health Sciences, University of the Witwatersrand, Johannesburg, South Africa; 4 Research Triangle Institue (RTI) International, 6110 Executive Boulevard, Rockville, USA

**Keywords:** Pneumonia, Streptococcus pneumoniae, child, sub-Saharan

## Abstract

**Background**: This study aimed to summarise the evidence on the impact of routine administration of 10-valent and 13-valent pneumococcal conjugate vaccines on pneumonia in children under five years of age in sub-Saharan Africa.

**Methods:** A systematic search of the literature was conducted including primary research reporting on the impact of 10- or 13-valent pneumococcal vaccines on childhood pneumonia in a sub-Saharan African country. Case-control, cohort, pre-post and time-series study designs were eligible for inclusion. Thematic narrative synthesis was carried out to summarise the findings.

**Results:** Eight records were included in the final analysis, 6 records were pre-post or time-series studies, 1 was a case-control study and 1 report combined pre-post and case-control studies. Vaccine impact on clinical pneumonia measured as percentage reduction in risk (%RR) was mostly non-significant. The reduction in risk was more consistent in radiological and pneumococcal pneumonia.

**Conclusions:** Evidence of the positive impact of routine infant pneumococcal vaccination on clinical pneumonia incidence in sub-Saharan Africa is inconclusive. Ongoing surveillance and further research is required to establish the long term trend in pneumonia epidemiology and aetiology after PCV introduction.

**PROSPERO registration**: CRD42019142369 30/09/19

## Introduction

Pneumonia is one of the leading causes of childhood deaths globally, particularly in sub-Saharan Africa ^1^. Annually over a 100 million cases of pneumonia are reported in children less than five years of age, mostly in poor countries in Africa and Asia ^2– 4^.

*Streptococcus pneumoniae* is the major cause of childhood pneumonia deaths and is the leading cause of vaccine-preventable child deaths globally ^5– 7^. Pneumococcal pneumonia causes between 1 and 4 million episodes of pneumonia in Africa yearly ^8^. There are currently about 100 known serotypes of *S. pneumoniae,* characterised by the polysaccharide capsule antigen ^9^. These serotypes differ in carriage potential and propensity to cause invasive disease including pneumonia, otitis media and meningitis ^10, 11^, with the 13 most common serotypes accounting for 70 – 75% of invasive pneumococcal disease globally ^8, 11, 12^.

Pneumococcal conjugate vaccines (PCV) have been licensed since 2000; previous polysaccharide-based vaccines were found to have poor immunogenicity in children ^8^. An initial 7-valent PCV included serotypes 4, 6B, 9V, 14, 18C, 19F and 23F providing cover against 67% of disease-causing serotypes. The 10-valent and 13-valent PCV include in addition to the 7-valent serotypes 1, 5 and 7F; and 1, 3, 5, 7F, 19A, and 6A. The 10-valent vaccine covers 70% - 84% while the 13-valent vaccine covers about 74% - 88% % of invasive disease-causing serotypes ^8, 13, 14^. Sub-Saharan African countries like South-Africa and The Gambia introduced the 7-valent PCV into routine infant vaccination schedule in 2009 ^15, 16^. Many countries in sub-Saharan Africa through the support of Gavi, the vaccine alliance, have incorporated the 10- or 13-valent PCV into their expanded program of immunization (EPI) schedules. The vaccines are usually administered as three doses in early infancy (3 + 0 schedule), or two doses in early infancy plus a booster in late infancy (2 + 1 schedule). PCV vaccination has been associated with a significant decline in invasive pneumococcal disease incidence globally and at individual country level ^9, 15, 16^. While there is evidence of effectiveness against invasive pneumococcal disease, the impact of vaccination on clinical and radiological pneumonia remains unclear ^16^.

### Objectives

This review aims to summarise the existing evidence on the impact of routine administration of pneumococcal conjugate vaccines on clinical pneumonia, radiological pneumonia and pneumococcal pneumonia in children under five years of age in sub-Saharan Africa.

## Methods

### Study protocol

The systematic review protocol was developed in accordance with the PRISMA-P guidelines ^17^, and registered with PROSPERO on 30 September 2019 ( CRD42019142369).

### Eligibility criteria

We included primary, individual and population-based studies conducted in sub-Saharan Africa evaluating 10-valent or 13-valent PCV impact in children published in English since 1 January 2010. This time was chosen because the earliest countries in this region to introduce PCV into routine infant vaccine schedule did so in 2009. The eligibility criteria are detailed in Table 1 below.

**Table 1.** PICO framework for formulating the review question.

Concept	Elaboration
Population of interest	Children between 1 – 59 months of age from any of the 46 countries in sub-Saharan Africa.
Intervention	Routine immunization with 10- or 13-Valent PCV
Comparison (or control)	Control group (either contemporary or historical)
Outcomes	Clinical pneumonia, radiological pneumonia or pneumococcal pneumonia
Study type	Interrupted time-series, pre-post studies, case control and cohort

Studies with invasive pneumococcal disease (IPD) as outcome were considered for inclusion if pneumonia cases were reported separately.

The study designs eligible for inclusion were pre-post quasi-experimental, interrupted time series, Cohort, and Case-control studies. For pre-post studies and interrupted time series, we included only studies where the final outcome assessment was made at least 3 years after vaccine introduction.

### Information sources

We conducted a search of peer-reviewed and grey literature relating to the study question. PubMed search was first conducted on 16 July 2019, with final search on 31 July 2019. Scopus search was conducted on 20 Jul 2019, Embase (Ovid) was searched on the 29 Jul 2019. Other peer-review sources include Africa-Wide Information (29 July 2019) and African Index Medicus (24 July 2019). Grey literature sources include OpenGrey (20 July 2019), and ProQuest Dissertation & theses global (20 July 2019), London School of Hygiene and Tropical Medicine research online (22 July 2019) and University of Edinburgh library (28 July 2019).

### Search strategy

The search strategy combined the key concepts of the research question and was based on the PICOS framework. The three components or concepts were: population of interest (children below 5 years of age), intervention being investigated (pneumococcal conjugate vaccine) and the outcome of interest (pneumonia). The Boolean operators AND, and OR were used to combine the search concepts.

Further details of the database search strategy and date of searches can be found as extended data ^18^.

### Study selection process

**Figure 1.**
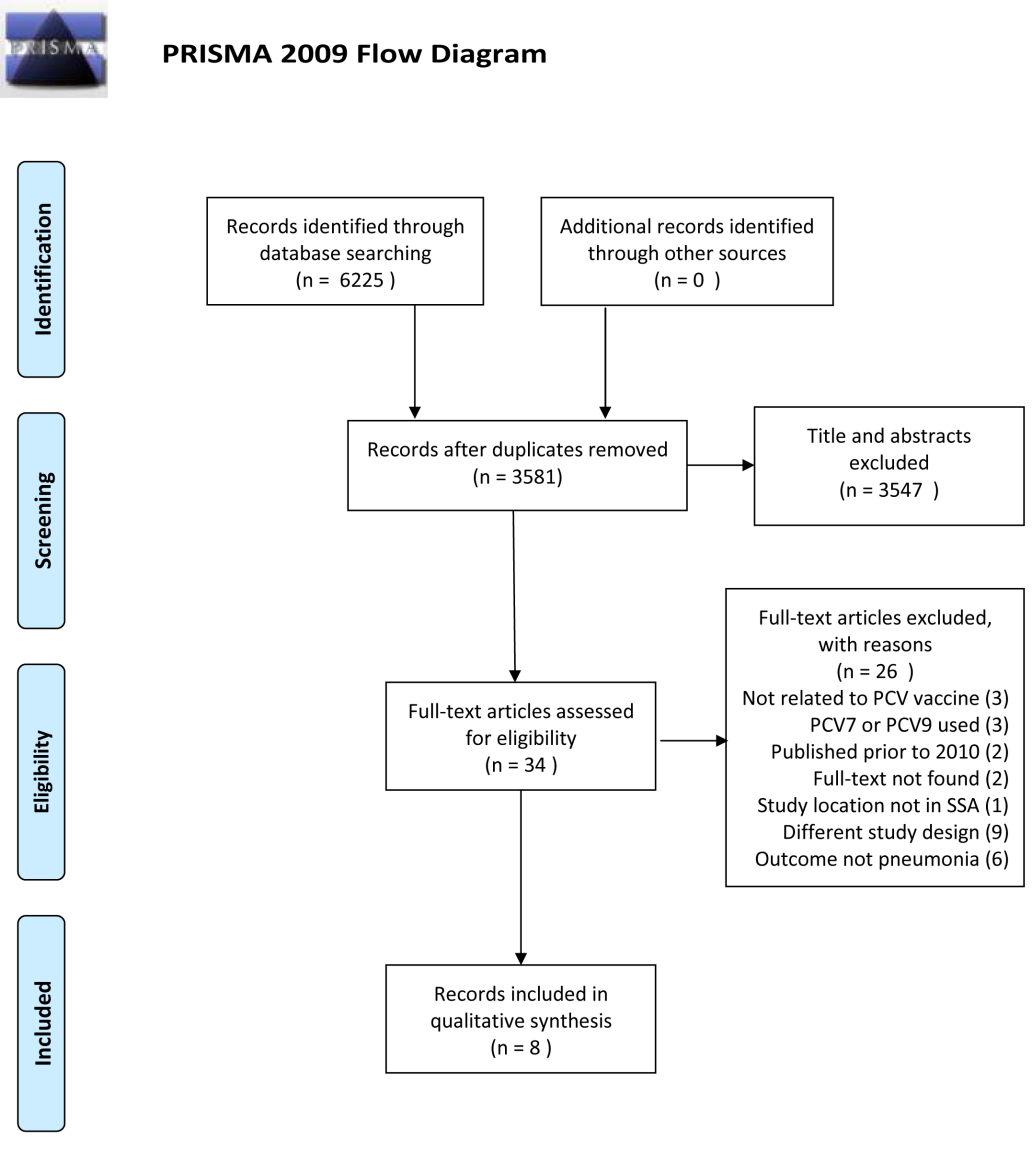
PRISMA flow diagram showing review selection process.

Two members of the review team conducted the database screening independently. Reading through the titles and abstracts of the search results we identified records to be included in the full-text screening based on the eligibility criteria. Records for which there was uncertainty or disagreement about eligibility during the title and abstract screening were included for full-text screening. The second stage of the screening involved reading the full-text of the records to determine if they were eligible for inclusion. Finally, we searched through the references of eligible papers for other relevant publications that could be included in the review. The PRISMA flow diagram for the study selection procedure is shown in Figure 1 in the result section.

### Data collection process and data items

The following information was collected for each included record: year of publication, study location and country, study setting (hospital-based or population-based), study design, study aim (to assess impact or effectiveness), data sources (clinical or laboratory), study population description, HIV status of participants, type of PCV in current use (PCV 10 or PCV13), year PCV7 introduced, year PCV10 or PCV13 introduced, reported coverage during PCV10 or PCV13 period, baseline and post-vaccination periods (for pre-post and interrupted time-series), outcome definition, outcome measure and confidence interval if reported. The information was collected directly into an extraction form in Excel by one member of the review team and crosschecked by a second member ^19^. Disagreement was resolved by consensus after discussion with another member of the review team. No additional information was sought from investigators or authors.

### Risk of bias in individual studies

 We assessed the quality of case-control studies assessing vaccine effectiveness, we used the National Heart, Lung and Blood Institute Quality assessment tool for case-control studies. We adapted the National Heart, Lung and Blood Institute Quality Assessment Tool for Before-After studies with No Control Group to assess quality in pre-post and interrupted time series analyses ^20^. The study quality assessment tools are available at https://www.nhlbi.nih.gov/health-topics/study-quality-assessment-tools and available as extended data ^21^.

### Summary measures

The primary outcomes of interest in this review were clinical pneumonia and radiological pneumonia. The secondary outcome was pneumococcal pneumonia. In pre-post and interrupted time-series studies comparing outcome incidence before and after PCV introduction, measures were presented as percentage reduction in incidence and incidence ratios. Where possible incidence ratios were converted to percentage reduction in incidence: *% reduction in risk = (1 – aRR) X 100%;* where aRR is the adjusted Risk/Rate ratio for post- and pre-vaccination periods ^22^. In case-control studies we presented adjusted vaccine effectiveness (aVE) as reported by the authors. When adjusted odd Ratios (aOR) were presented we estimated aVE as: *adjusted vaccine effectiveness = (1 – aOR) X 100%* ^19^ *;* where aOR = adjusted odd ratio. All calculations were made in Microsoft Excel 2016, and graphical presentations were performed in Stata 13 ^23^.

### Synthesis of results

Planned quantitative synthesis could not be conducted in this review due to variation in included studies. We therefore, present a narrative synthesis of the impact of PCV on childhood clinical pneumonia, radiological pneumonia and pneumococcal pneumonia.

## Results

### Study selection

The database search produced 3581 distinct titles, from which we identified 34 relevant records for the full-text review. We excluded a further 26 records after full-text assessment. Eight records were included in the review as shown in Figure 1 below.

### Study characteristics

Four of the included studies were conducted in South Africa (23 – 26), two were conducted in Kenya ^24, 25^, and one each from Rwanda ^26^, and The Gambia ^27^. There were 7 studies with pre-post or interrupted time-series design and two case-control studies (Mackenzie *et al.* included report on a case-control study). Most of the study locations had 7-valent PCV introduced before transitioning to the 13-valent PCV except in Kenya where the 10-valent PCV was used without a 7-valent PCV period. Table 2 below shows the characteristics of articles included in the review.

**Table 2.** Characteristics of studies included in review.

Author	Country	Study design	Data sources	Population	PCV	Year PCV7 introduced	Year PCV 10 or 13 introduced	Dosing schedule	Baseline period	Post- intervention period
**Von Mollendorf *et al.*, 2017** **** ^30^	South Africa	Pre-post	Laboratory surveillance	< 60 months	13	2009	2011	Week 6 and 14, with booster at 9 months (2 + 1 schedule)	2005 – 2008	2012 - 2013
**Mackenzie *et al.*,** **2017** ^31^	URR, The Gambia	Pre-post	Population surveillance	2 to 59 months	13	2009	2011	Month 2, 3 and 4 (3 + 0 schedule)	2008 – 2010	2014 - 2015
**Mackenzie *et al.*,** **2017** * ^31^	URR, The Gambia	Case- control	Clinical	3 to 59 months	13	2009	2011	As above	NA	NA
**Hammit *et al.*, 2019** ^24^	Kilifi, Kenya	Pre-post	Clinical surveillance	< 60 months	10	NA	2011	Week 6, 10 and 14 (3 + 0 schedule)	1999 – 2010	2012 - 2016
**Gatera *et al.*,** **2016** ^25^	Rwanda (five districts)	Pre-post	Clinical and laboratory surveillance	< 60 months	13	2009	2011	Week 6, 10 and 14 (3 + 0 schedule)	2002 – 2009	2012
**Silaba *et al.*, 2019** ^32^	Kilifi, Kenya	ITS	Population surveillance	2 to 59 months	10	NA	2011	Week 6, 10 and 14 (3 + 0 schedule)	2002 – 2011	2011 - 2015
**Mahdi *et al.*, 2015** ^33^	Soweto, Cape town, KwaZulu- Natal. South Africa	Case- control	Clinical	2 to 42 months	13	2009	2011	Week 6 and 14, with booster at 9 months (2 + 1 schedule)	NA	NA
**Solomon *et al.*, 2017** ^34^	Soweto, South Africa	Pre-post	Population surveillance	< 60 months	13	2009	2011	Week 6 and 14, with booster at 9 months (2 + 1 schedule)	2006 – 2008	2014
**Tempia *et al.*, 2015** ^35^	Soweto, South	Pre-post	Laboratory surveillance	< 24 months	13	2009	2011	Week 6 and 14, with booster at 9 months (2 + 1 schedule)	2009 -	2011 – 2012

NA: Not applicable, * Reported along with the pre-post study.

### Risk of bias within studies

One of the seven population-based studies were considered to be of poor quality with significant risk of bias. None of the population-based studies were blinded hence there is the possibility of bias in the findings. The two case-control reports were graded as having good quality. Details on the study quality assessment are available as extended data ^21^.

### Result of individual studies

There was variation in the definition of clinical and radiological pneumonia, and in the method of identification of pneumococcal pneumonia among the included studies. Some studies applied the WHO standards for identification of radiological pneumonia and clinical pneumonia ^25, 27, 28^. One study based pneumonia diagnosis on ICD-10 coding from clinical notes ^29^. Studies reporting on pneumococcal pneumonia were based on culture with occasional confirmation by polymerase chain reaction (PCR). Table 3 below summarises the findings of the included studies.

**Table 3.** Summary of effect reported in studies included in the review.

Study	population	Clinical pneumonia	Radiological pneumonia	Pneumococcal pneumonia
		Percent rate reduction (95% CI)	Percent rate reduction (95% CI)	Percent rate reduction (95% CI)
**Von Mollendorf *et al.*,** **2017** ^30^	< 60 months	--	--	65 (64 – 67)
	HIV positive	--	--	51 (49 – 55)
	12 – 59 months	--	--	69 (67 – 71)
**Mackenzie *et al.*,** **2017** ^31^	2 – 11 months	2 (-4 – 8)	23 (7 – 36)	--
	12 – 23 months	-6 (-15 – 2)	29 (12 - 42)	--
	24 – 59 months	-7 (-18 – 2)	22 (1 - 39)	--
**Hammit *et al.*, 2019** ^24^	< 60 months	--	--	85 (66 – 93)
**Gatera *et al.*, 2016** ^25^	< 60 months	15 (Not reported)	--	--
**Silaba *et al.*, 2019** ^32^	2 – 59 months	27 (3 – 46)	48 (14 – 68)	--
	2 – 11 months	--	27 (-36 – 61)	--
	12 – 23 months	--	46 (-10 – 73)	--
	24 – 59 months	--	11 (-69 – 53)	--
**Solomon *et al.*,** **2017** ^34^ **	HIV infected	33 (6 -52) ¶	--	--
	HIV uninfected or unconfirmed	39 (24 – 50) ¶	--	--
	3 – 11 months	39 (10 – 57) ¶	--	--
	12 – 23 months	15 (-37 – 42) ¶	--	--
	24 – 59 months	45 (17 – 62) ¶	--	--
**Tempia *et al.*, 2015** ^35^	HIV uninfected	--	--	66.8 (43.8 – 81.3)
		--	--	64.0 (52.6 – 81.2)
		Adjusted vaccine effectiveness (95% CI)	Adjusted vaccine effectiveness (95% CI)	Adjusted vaccine effectiveness (95% CI)
**Mackenzie *et al.*,** **2017** * ^31^	3 – 59 months	--	28 (-23 – 58)	--
**Mahdi *et al.*, 2015** ^33^	Hospital controls	--	20.0 (-9.3 – 41.6)	--
	Community controls	--	32.1 (4.6 – 51.6)	--

*Based on WHO clinical classification of severe and very severe pneumonia, ** pneumonia hospitalization based on ICD-10 coding, ¶ 50% credible interval based on Bayesian methods (negative values indicate an increase in incidence), € Identification based on polymerase chain reaction, ± pneumonia classified based on WHO criteria or based on abnormal CXR plus C-reactive protein >40mg/L.

### Synthesis of results

**Figure 2.**
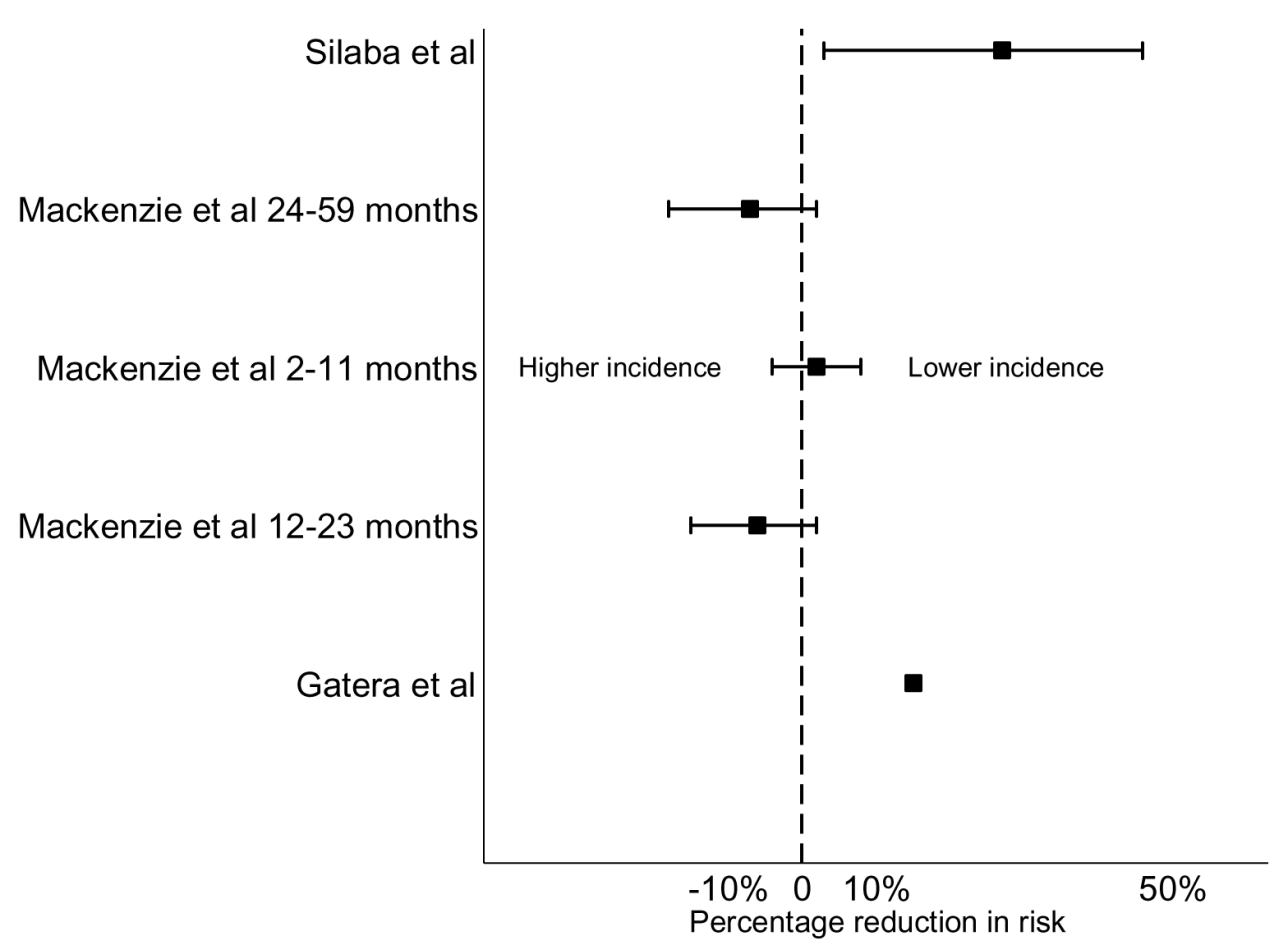
Forest plot showing the percentage risk reduction in clinical pneumonia incidence in included studies and population.

***Impact on clinical pneumonia***. Four population-based studies measured the impact of 10- or 13-valent vaccine on clinical pneumonia with inconsistent findings. Silaba *et al.* reported a decline in hospitalization for severe or very severe pneumonia based on WHO clinical definition ^25^. Mackenzie *et al.* showed no significant decline in clinical pneumonia incidence in all under-five age groups three years after 13-valent PCV introduction ^27^. Solomon *et al.* estimated a significant decline in pneumonia hospitalizations in infants and children between 24 – 59 months based on Bayesian methods. This effect was also found among HIV infected and HIV uninfected children ^29^. However, these figures were estimated around a 50% credible interval. Figure 2 graphically displays the reported reduction in clinical pneumonia incidence.

***Impact on radiological pneumonia***. Two impact studies evaluated radiological pneumonia as outcomes ^25, 27^. Mackenzie *et al.* reported a decline in WHO defined radiological pneumonia in all age groups with decline in the range of 22 – 29%. This was most pronounced in the 12 to 23-month age group. Silaba *et al.* also reported a 48% decline in radiological pneumonia in the entire under-five population. A similar age-related trend was observed, with children between 12 to 23-month age experienced the largest reduction in radiological pneumonia ^25^.

The case-control study reported by Mackenzie *et al.* using community controls showed a vaccine effectiveness of about 28%, however, this did not reach statistical significance. Mahdi *et al.* reported adjusted vaccine effectiveness measures using both community and hospital controls ^28^. Vaccine effectiveness was significant with community controls (aVE 32.1%, 95% CI 4.6% - 51.6%), but not with hospital controls (aVE 20%, 95% CI -9.3% – 41.6%).

**Figure 3.**
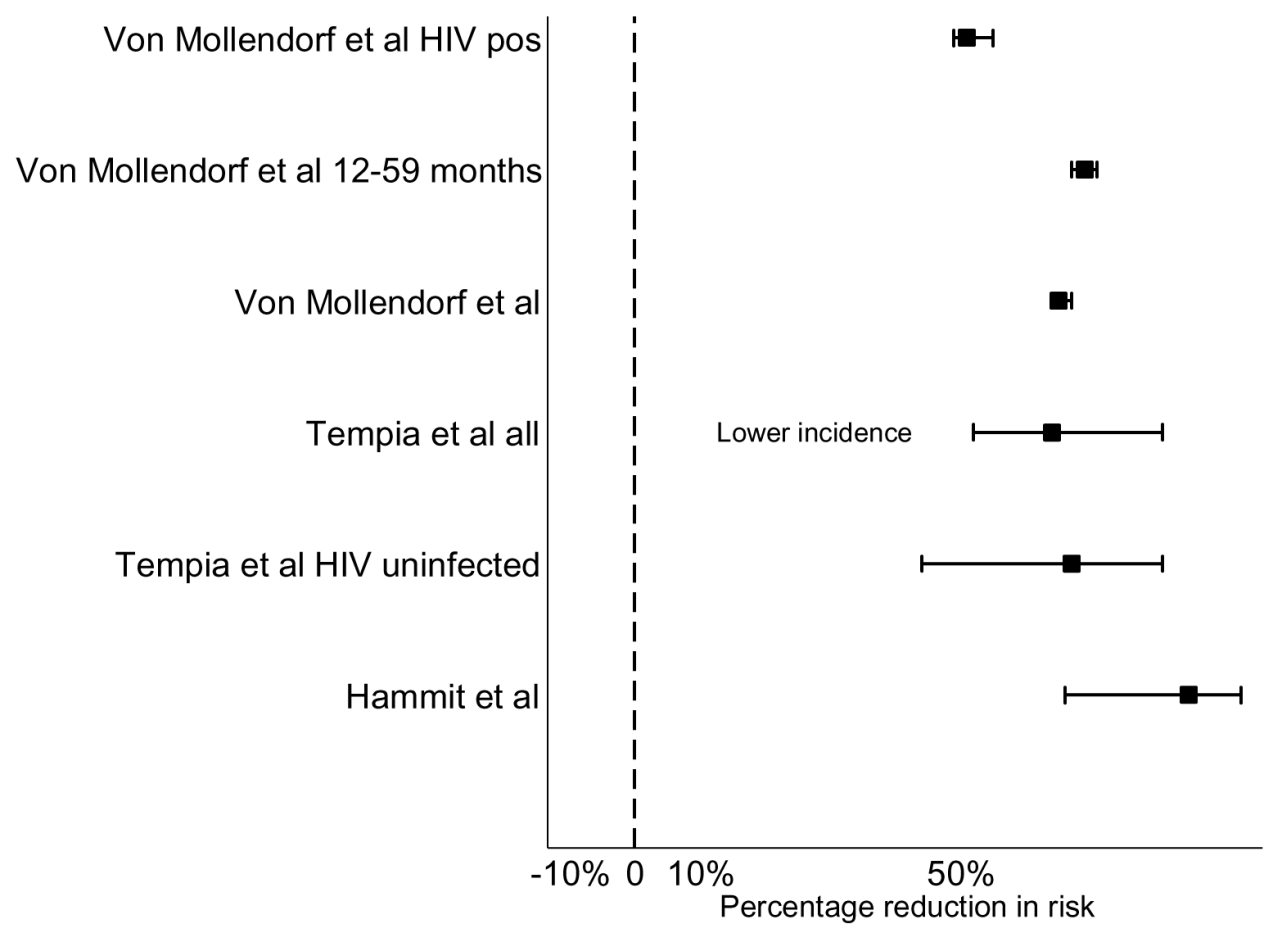
Forest plot showing the percentage risk reduction in pneumococcal pneumonia incidence in included studies and population.

***Impact on pneumococcal pneumonia***. All three studies that reported on pneumococcal pneumonia were based on microbiological diagnosis with or without PCR confirmation ^24, 31, 36^. Included studies consistently showed decline in cases of pneumococcal pneumonia after vaccine introduction irrespective of method of pneumococcal identification. Figure 3 shows the reduction in pneumococcal pneumonia incidence reported from included studies.

## Discussion

### Summary of evidence

This review set out to answer the question: has the introduction of pneumococcal conjugate vaccine resulted in a decline in childhood pneumonia? From our review, we can summarise that the population impact of pneumococcal vaccination on pneumonia depends largely on how pneumonia is classified. We observed overall that when the outcome is clinical pneumonia, the impact tends to be modest at best. We see this in the reports by Mackenzie *et al.*, Silaba *et al.*, and Solomon *et al.* However, it is important to note that the severity of pneumonia differed in these studies. Mackenzie *et al.* evaluated clinical pneumonia at the population level and showed results that were not statistically significant in all age groups. Silaba *et al.*, however, looked at clinical pneumonia hospitalizations (i.e. severe or very severe pneumonia) and showed significant difference in incidence after vaccine introduction. We also see a subtle decline in hospitalizations for clinical pneumonia based on ICD-10 coding. Clinical pneumonia is a non-specific outcome and therefore may not be ideal for evaluating disease-specific vaccine impact. There was a more positive but still modest impact observed with radiological pneumonia as an outcome. This is likely because radiological pneumonia is more specific for pneumococcal disease. Overall, there was a consistent decline in pneumococcal pneumonia cases in the post-vaccine period in reported impact studies ^24, 31, 36^. Two studies that reported on vaccine effect in HIV infected children showed that pneumococcal vaccine had similar impact as with HIV uninfected children ^29, 31^.

The overall trend in the findings from this review is similar to reports from other parts of the world. Meta-analyses from other regions have found modest decline in clinical and radiological pneumonia hospitalizations. A meta-analysis using global data from 12 pre-post and time series studies produced a pooled reduction in hospitalization rates for clinical pneumonia of 17% and 9% in the under 24 months and 24 – 59 months’ age groups. However, they reported a more significant decline in hospitalization rates due to radiological pneumonia of 31% and 24% in the same age groups ^37^. A randomised placebo-controlled trial on 9-valent vaccine showed vaccine effectiveness of 37% based on per-protocol population ^22^. Overall, it appears that pneumococcal vaccination has its greatest impact in preventing the more severe forms of pneumonia for which children are likely to be hospitalised. This is probably due to the finding that bacterial pathogens are more likely to cause severe pneumonia ^30^. The minimal impact on clinical pneumonia might suggest an increase in other forms of pneumonia or may be due to serotype replacement ^32– 34^. We observed an age-related trend in vaccine effect, with the 12 to 23-month age group experiencing the greatest benefit. This might be due in part to a greater proportion of children under 12 months having not completed their vaccination schedules; and potentially waning immunity in the older age groups ^35^.

### Limitations

This review has some limitations that have to be considered when interpreting our findings. First was the inconsistency in the definition of pneumonia outcomes between studies, which made combining the impact measured between studies impractical. While some studies used comparable methods for outcome ascertainment, this was not consistent across studies. The WHO standardised definition of clinical and radiological pneumonia is markedly helpful in this situation as it ensures consistency across studies ^38^. Studies conducted in locations with a functional health and demographic surveillance system like in the Upper River region of The Gambia and in Kilifi, in Kenya, were particularly robust as they combined consistent pneumonia surveillance methods with up-to-date population information ^24, 27^. Some studies relied on routine clinical data which is usually of variable quality ^26, 29^.

It is also important to note that while we set out to evaluate the impact of 10-valent and 13-valent vaccines, all of the study locations except in Kenya had a period of 7-valent vaccine use. Therefore, is it impossible to separate the effect of the 7-valent from the 10- and 13-valent vaccines since the 7-valent PCV might have influenced the pre-existing disease trend. However, because of the short time lapse between the 7-valent and 10- or 13-valent vaccine roll-out in these countries, it is unlikely that a significant change in pneumonia trend would be demonstrable.

Like all time-trend studies - including pre-post studies, phenomena such as regression to the mean, seasonality, trend, and history bias have to be considered in the analysis. By including a control outcome and conducting sensitivity analysis, some of the included studies considered the impact of history bias and trend on their results ^39^.

Publication bias is a potential limitation of this review. However, due to the small number of reports in each outcome category, we did not formally assess for publication bias using a funnel plot ^40^. We are therefore unable to comment on publication bias in this review. Finally, due to limited resources we were unable to include studies published in other languages; hence, language bias cannot be ruled out in our review.

## Conclusion

To the best of our knowledge this is the first systematic attempt at summarising the impact and effectiveness of routine pneumococcal vaccination on childhood pneumonia from this region. The 10- and 13-valent PCV use as part of infant immunization is effective in preventing the more severe forms of childhood pneumonia. There appears to be a smaller effect on clinical pneumonia especially when all severity spectra are included. There is the need for consistency in pneumonia definition for the purpose of disease surveillance and the WHO clinical definitions provide an appropriate option ensuring ease of implementation and reproducibility. One major issue encountered was that few studies had applied comparable pneumonia definitions in estimating disease burden prior to pneumococcal vaccine introduction hence making trend analysis difficult. There is the need for the generation of updated information on the causes of pneumonia in this region in this era of extensive pneumococcal vaccine use. Ongoing surveillance is needed to investigate the long-term trend in childhood pneumonia in the PCV era in sub-Saharan Africa.

## Data availability

### Underlying data

All data underlying the results are available as part of the article and no additional source data are required.

### Extended data

This project contains the following extended data:

Figshare: Detailed search https://doi.org/10.6084/m9.figshare.12656309.v2 ^18^

Figshare: Data extraction form https://doi.org/10.6084/m9.figshare.12608264 ^19^

Figshare: Quality assessment tool https://doi.org/10.6084/m9.figshare.12608264 ^21^

### Reporting guidelines

Figshare: PRISMA checklist for ‘Estimating the impact of pneumococcal conjugate vaccines on childhood pneumonia in sub-Saharan Africa: A systematic review’. https://doi.org/10.6084/m9.figshare.12608672.v2 ^41^

Data are available under the terms of the Creative Commons Attribution 4.0 International license (CC-BY 4.0).
