# Supplementary material for: Estimating the impact of pneumococcal conjugate vaccines on childhood pneumonia in sub-Saharan Africa: A systematic review
Source: F1000Res. 2020 Nov 9;9:765. Originally published 2020 Jul 23. [Version 2] doi: 10.12688/f1000research.25227.2 (PMC7713889; doi:10.12688/f1000research.25227.2)
Supplement: Peer review history

# Peer review: Reviewer response for version 2

**DOI:** 10.5256/f1000research.30623.r74452

**Published:** 2020-12-01

**Authors:** Dodi Safari

**Affiliations:** 1 Molecular Bacteriology Unit, Eijkman Institute for Molecular Biology, Jakarta, Indonesia

I have reviewed the revision and the authors have answered all the queries satisfactorily

Are the rationale for, and objectives of, the Systematic Review clearly stated?

Partly

Is the statistical analysis and its interpretation appropriate?

I cannot comment. A qualified statistician is required.

Are sufficient details of the methods and analysis provided to allow replication by others?

Yes

Are the conclusions drawn adequately supported by the results presented in the review?

Partly

Reviewer Expertise:

Medical microbiology

I confirm that I have read this submission and believe that I have an appropriate level of expertise to confirm that it is of an acceptable scientific standard.

# Peer review: Reviewer response for version 1

**DOI:** 10.5256/f1000research.27840.r73233

**Published:** 2020-11-02

**Authors:** Dodi Safari

**Affiliations:** 1 Molecular Bacteriology Unit, Eijkman Institute for Molecular Biology, Jakarta, Indonesia

In this systematic review with the title “Estimating the impact of pneumococcal conjugate vaccines on childhood pneumonia in sub-Saharan Africa”, the authors showed evidence of the impact of pneumococcal conjugate vaccines (PCV10 and PCV13) in Kenya, Gambia, Rwanda, and South Africa is inconclusive based on clinical pneumonia incidence.

The authors may need to elaborate how the impact of the first introduction of PCV7 vaccine on pneumonia incidence in this systematic review since most of the study location had PCV7 introduced before PCV10/PCV13, except in Kenya (page 5). 
The authors need to add the information on the immunization doses and schedule in each location and to discuss further how these differences had an impact on the outcomes of PCV10 and PCV13 vaccination.  
The authors need to discuss further the health and economic status in each location that can affect the outcomes of PCV10 and PCV13 vaccination. 
4. Please add the new update of pneumococcal polysaccharide capsule (Introduction part):
  https://mbio.asm.org/content/11/3/e00937-20 ^1^

Are the rationale for, and objectives of, the Systematic Review clearly stated?

Partly

Is the statistical analysis and its interpretation appropriate?

I cannot comment. A qualified statistician is required.

Are sufficient details of the methods and analysis provided to allow replication by others?

Yes

Are the conclusions drawn adequately supported by the results presented in the review?

Partly

Reviewer Expertise:

Medical microbiology

I confirm that I have read this submission and believe that I have an appropriate level of expertise to confirm that it is of an acceptable scientific standard, however I have significant reservations, as outlined above.

## References

[1] : A New Pneumococcal Capsule Type, 10D, is the 100th Serotype and Has a Largecps Fragment from an Oral Streptococcus. *mBio*.2020;11(3) : 10.1128/mBio.00937-20 10.1128/mBio.00937-20 PMC724015832430472

# Response

**Published:** 2020-11-03

**Authors:** chukwuemeka Onwuchekwa

**Affiliations:** Institute of Tropical Medicine, Antwerp, Belgium

Thank you for reviewing our work and for the constructive comments and suggestions. We have carefully considered your comments and address them below:

 1. As stated in our original submission:

*“it impossible to separate the effect of the 7-valent from the 10- and 13-valent vaccines since the 7-valent PCV might have influenced the pre-existing disease trend”*.

 We have gone further to stress that separating the effect of the 7-valent from subsequent 13-valent vaccination is further compounded by the short time lapse between the two vaccines (2009 and 2011). We therefore added the statement:

*“However, because of the short time lapse between the 7-valent and 10- or 13-valent vaccine roll-out in these countries, it is unlikely that a significant change in pneumonia trend would be demonstrable”*

 2. Thank you for drawing our attention to the influence of dosing schedule, this comment was very helpful. We have included the dosing schedule used in each country as described by the authors in Table 2 (Description of included studies). In the introduction section, we have also included a sentence describing the current vaccine schedule:

*“The vaccines are usually administered as three doses in early infancy (3 + 0 schedule), or two doses in early infancy plus a booster in late infancy (2 + 1 schedule)”*

 While there is evidence that pneumococcal vaccine schedules which include a booster (e.g. 2 + 1 or 3 + 1 schedules) are associated with a more robust immune response, it has not been shown that these schedules have any effect of carriage or indeed pneumonia incidence(1). We have therefore not explored this further as we considered this to be outside the boundaries of our review, and we have too few studies to make any type of comparison.

 3. Thank you for this comment. We agree with your statement about the influence of Health and economic situation of the populations and how this could have influence childhood pneumonia and indeed uptake of vaccination. We also indicate here that the uptake of vaccination in all the reports were above 90% in the relevant population. Furthermore, the population based impact studies compared populations before and after vaccine roll-out, with a relatively short time lapse between the baseline and post-vaccination periods. It is our belief that socioeconomic variables are unlikely to have changed significantly at population-level, and is likely to have minimal influence on the observed findings. More importantly, most of the population based studies (time-series and before-after studies) included a control group or control condition, which would allow for assessment of the influence of historical bias (including changes in socioeconomic conditions)(2)

 4, Thank you for drawing our attention to the most recent information of pneumococcal serotypes. We have noted this comment and made changes to the manuscript to reflect this, also included is the reference you suggested.

 References

 1.          Whitney CG, Goldblatt D, O’Brien KL. Dosing schedules for pneumococcal conjugate vaccine: considerations for policy makers. Pediatr Infect Dis J. 2014 Jan;33 Suppl 2:S172-81.

 2.          Bernal JL, Cummins S, Gasparrini A. The use of controls in interrupted time series studies of public health interventions. Int J Epidemiol. 2018;47(6):2082–93.

# Peer review: Reviewer response for version 1

**DOI:** 10.5256/f1000research.27840.r67910

**Published:** 2020-10-06

**Authors:** Blandina Theophil Mmbaga

**Affiliations:** 1 Kilimanjaro Christian Medical Center, Kilimanjaro Clinical Research Institute, Moshi, Tanzania

Thank you for the opportunity to review this manuscript. The author works on the important topic taking into account pneumonia still remains as one of the major causes of childhood morbidity and mortality. Assessing the impact of vaccines while differentiating clinical, radiological outcome support the evidence on the vaccine impact. 

 The manuscript is written in good English language.

 The methodology is clear on how the review was conducted and how they reached final articles for inclusion. 

 No major specific comments and would recommend the manuscript acceptance.

Are the rationale for, and objectives of, the Systematic Review clearly stated?

Yes

Is the statistical analysis and its interpretation appropriate?

Yes

Are sufficient details of the methods and analysis provided to allow replication by others?

Yes

Are the conclusions drawn adequately supported by the results presented in the review?

Yes

Reviewer Expertise:

Paediatric infectious disease

I confirm that I have read this submission and believe that I have an appropriate level of expertise to confirm that it is of an acceptable scientific standard.

# Response

**Published:** 2020-10-06

**Authors:** chukwuemeka Onwuchekwa

**Affiliations:** Institute of Tropical Medicine, Antwerp, Belgium

Thank you for the favourable review.
